# DiSCIoser: unlocking recovery potential of arm sensorimotor functions after spinal cord injury by promoting activity-dependent brain plasticity by means of brain-computer interface technology: a randomized controlled trial to test efficacy

**DOI:** 10.1186/s12883-023-03442-w

**Published:** 2023-11-21

**Authors:** Emma Colamarino, Matteo Lorusso, Floriana Pichiorri, Jlenia Toppi, Federica Tamburella, Giada Serratore, Angela Riccio, Francesco Tomaiuolo, Alessandra Bigioni, Federico Giove, Giorgio Scivoletto, Febo Cincotti, Donatella Mattia

**Affiliations:** 1 Department of Computer, Control, and Management Engineering “Antonio Ruberti”, Sapienza University of Rome, Via Ariosto, 25, 00185 Rome, Italy; 2 IRCCS Fondazione Santa Lucia, Via Ardeatina, 306, 00179 Rome, Italy; 3 Department of Clinical and Experimental Medicine, University of Messina, Piazza Pugliatti, 1, 98122 Messina, Italy; 4 Museo Storico Della Fisica E Centro Studi E Ricerche Enrico Fermi, Via Panisperna, 89a, 00184 Rome, Italy

**Keywords:** EEG-based Brain-Computer Interface, Spinal cord injury, Hand functional sensorimotor recovery, Brain plasticity, Motor imagery, Neurorehabilitation

## Abstract

**Background:**

Traumatic cervical spinal cord injury (SCI) results in reduced sensorimotor abilities that strongly impact on the achievement of daily living activities involving hand/arm function. Among several technology-based rehabilitative approaches, Brain-Computer Interfaces (BCIs) which enable the modulation of electroencephalographic sensorimotor rhythms, are promising tools to promote the recovery of hand function after SCI. The “DiSCIoser” study proposes a BCI-supported motor imagery (MI) training to engage the sensorimotor system and thus facilitate the neuroplasticity to eventually optimize upper limb sensorimotor functional recovery in patients with SCI during the subacute phase, at the peak of brain and spinal plasticity. To this purpose, we have designed a BCI system fully compatible with a clinical setting whose efficacy in improving hand sensorimotor function outcomes in patients with traumatic cervical SCI will be assessed and compared to the hand MI training not supported by BCI.

**Methods:**

This randomized controlled trial will include 30 participants with traumatic cervical SCI in the subacute phase randomly assigned to 2 intervention groups: the BCI-assisted hand MI training and the hand MI training not supported by BCI. Both interventions are delivered (3 weekly sessions; 12 weeks) as add-on to standard rehabilitation care. A multidimensional assessment will be performed at: randomization/pre-intervention and post-intervention. Primary outcome measure is the Graded Redefined Assessment of Strength, Sensibility and Prehension (GRASSP) somatosensory sub-score. Secondary outcome measures include the motor and functional scores of the GRASSP and other clinical, neuropsychological, neurophysiological and neuroimaging measures.

**Discussion:**

We expect the BCI-based intervention to promote meaningful cortical sensorimotor plasticity and eventually maximize recovery of arm functions in traumatic cervical subacute SCI. This study will generate a body of knowledge that is fundamental to drive optimization of BCI application in SCI as a top-down therapeutic intervention, thus beyond the canonical use of BCI as assistive tool.

**Trial registration:**

Name of registry: DiSCIoser: improving arm sensorimotor functions after spinal cord injury via brain-computer interface training (DiSCIoser). Trial registration number: NCT05637775; registration date on the ClinicalTrial.gov platform: 05-12-2022.

## Background

Traumatic Spinal Cord Injury (SCI) represents a devastating condition for physical and social well-being [1]. It is increasingly recognised as a global health priority due to the complex and expensive medical treatment required [2]. Despite numerous neuroprotective and neuroregenerative strategies recently proposed [3], there is still no consistent approach to this complex and multifaceted clinical condition [4].

Over the last decade, the epidemiology of traumatic SCI has progressively changed with an increase in cervical lesions, particularly at C1-C4 levels [5]. Depending on the level and severity of injury [6], arm and hand functions are impaired to varying degrees. Reduced upper extremity ability compromises the completion of activities of daily living. Therefore, recovery of arm/hand function is highly valued by individuals with cervical lesions and is considered the most important factor in improving their quality of life [7–9].

Technological advances have led to the development of novel interventions aimed at improving upper arm function in individuals with SCI [10, 11], and advances in neuroimaging techniques have provided evidence of structural and functional reorganization of the brain after traumatic SCI in both animal models [12] and humans. The potential role of such remote brain reorganization in promoting sensorimotor functional recovery has been widely emphasised [13–15], but there is still a limited number of studies investigating the effects of arm and hand function training in individuals with cervical SCI.

Current rehabilitation approaches after traumatic SCI mainly consist of intensive training of lost or impaired function that is assumed to increase the activity-dependent plasticity of spared circuits and thus leading to functional improvements [16]. Recently, neuromodulatory interventions targeting the sensorimotor systems at various levels have been applied in humans with SCI in combination with training to improve functional recovery [17–19]. Neurological rehabilitation of SCI may also benefit from cognitive training based on motor imagery (MI) [20], which allows active stimulation of brain motor areas promoting brain plasticity associated with positive effects on motor performance [21–23].

In the effort of encouraging the top-down contribution of supraspinal sensorimotor signal in SCI rehabilitation, the Brain-Computer Interface (BCI) technology [24] may provide fundamental tools not only for the restoration but also for the recovery of sensorimotor function. The long history of BCI research in SCI has been substantially devoted to developing systems to control external devices to restore function [25–27]. However, recent findings indicate that non-invasive BCI training in combination with intensive rehabilitation may be useful for individuals with chronic SCI to recover gait [28] and arm function [29].

The main evidence for the efficacy of non-invasive BCIs as neurorehabilitation tools stems from clinical trials conducted on stroke population, in which BCI-assisted MI training was shown to be effective in promoting brain plasticity, resulting in improved hand motor function [30]. These promising findings corroborate the idea that a low-cost technique such as electroencephalography (EEG)-based BCIs can be exploited to optimize the delivery of MI based rehabilitation interventions beyond stroke.

Moreover, despite differences in the usual mechanism of injury between stroke (ischaemia) and SCI (trauma), rehabilitation approaches could successfully translate in similar recovery of sensorimotor functions between ischaemic and traumatic SCI [31]. Furthermore, in both stroke and SCI, neuroplasticity may be considered the key to overcoming the injury-induced loss of central nervous system tissue and the resulting sensorimotor deficits [16].

For this reason, it could be assumed that monitoring and modulating the central nervous system plasticity occurring after a SCI is a key factor in shaping clinically valuable top-down rehabilitation strategies with the aim to recover sensorimotor function after SCI. In the DiSCIoser study, we propose to use a goal-oriented action imagination training, controlled, and objectified by means of a BCI system. As such, the BCI based motor imagination training would engage the sensorimotor system and thus facilitate neuroplasticity to eventually optimize upper limb sensorimotor functional recovery in patients with SCI during the subacute phase when brain and spinal plasticity is at its peak.

### Aim and hypotheses

The “DiSCIoser'' study is a randomized controlled trial (RCT) designed to provide evidence for a significant improvement of hand sensorimotor function induced by a BCI-assisted MI training in patients with traumatic SCI.

Based on our previous findings on the efficacy of BCI-supported MI training in patients with stroke [30], we hypothesise that establishing a real-time contingency between the content of MI and an ecological feedback specifically designed to train MI in patients with SCI will boost the effect of MI training in engaging the sensorimotor system, promoting meaningful cortical sensorimotor plasticity and eventually maximizing recovery of hand function.

Accordingly, the aim of the RCT will be to determine whether the BCI-assisted MI (BCI-MI) training, administered by means of a BCI system fully compatible with a clinical setting, is superior to a non-BCI assisted MI (Control-MI) training in improving hand sensorimotor function outcomes in individuals with traumatic cervical SCI admitted to the ward “Centro Spinale” at Fondazione Santa Lucia for their standard rehabilitation care.

## Methods/design

A total cohort of 30 participants will be enrolled and randomized in a single centre, single blind RCT to investigate the efficacy of BCI-MI training for hand movements, delivered during the subacute phase of SCI (hospitalization period). The primary and secondary outcome measures collected from participants assigned to BCI-MI training will be compared with those of participants who receive an equivalent dose of MI training without BCI support (Control-MI).

All procedures conducted in this trial follow national institutional ethical standards and the Helsinki Declaration. The study protocol and related procedures were approved by the institutional review board: the Independent Ethical Committee of the Fondazione Santa Lucia (FSL), I.R.C.C.S., Rome, Italy (protocol CE/PROG.884 15-12-2020). The trial was registered on the clinicaltrials.gov in 05-12-2022 (trial registration number: NCT05637775).

### Study design

**Fig. 1:**
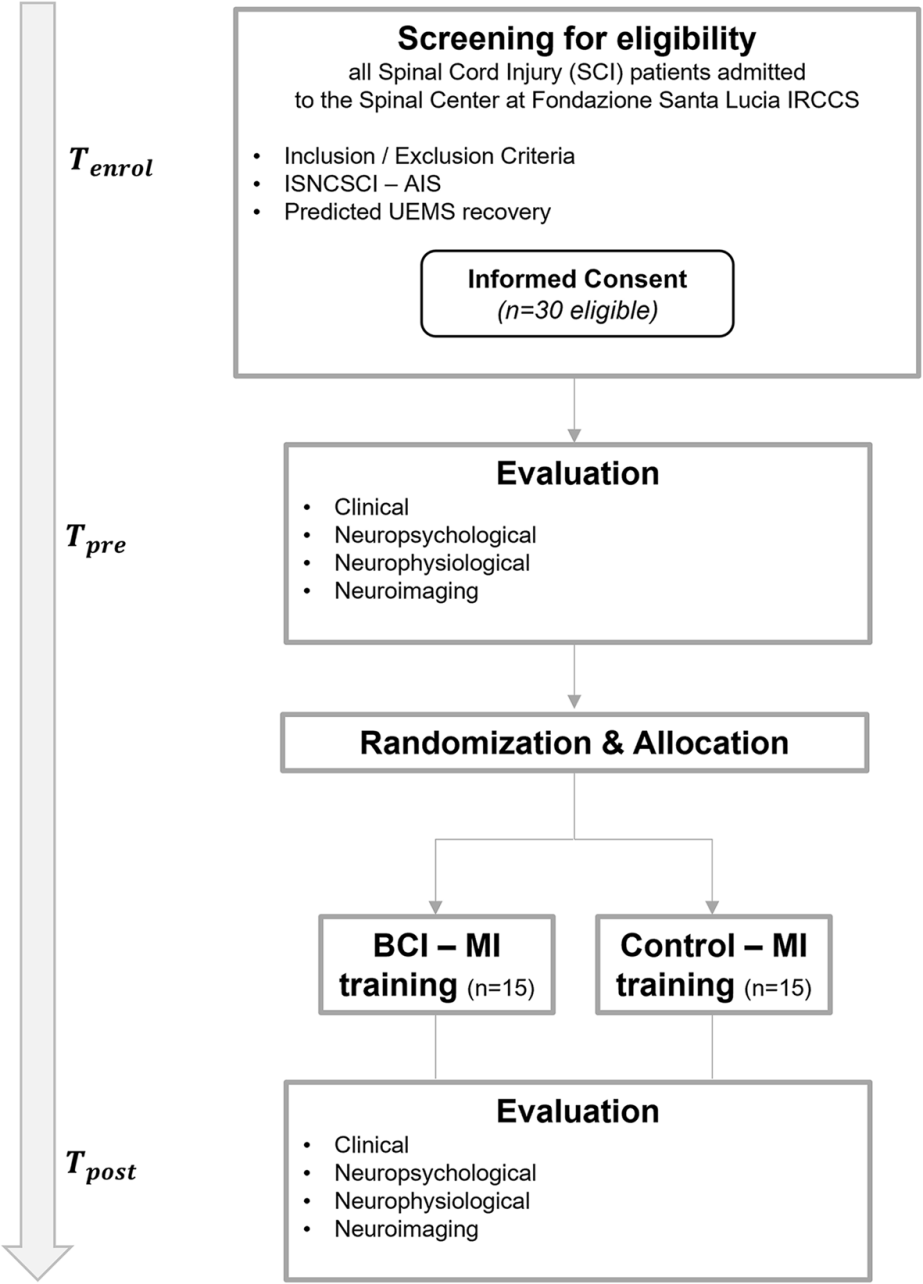
DiSCIoser Randomized Controlled Trial summary. All Spinal Cord Injury (SCI) patients admitted to the Spinal Unit at Fondazione Santa Lucia IRCCS will be screened (T enrol) for eligibility according to Inclusion/Exclusion criteria. The evaluation according to the International Standards for Neurological Classification of SCI (ISNCSCI) ASIA Impairment Scale (AIS) and the predicted mean Upper Extremity Motor Score (UEMS) recovery will be used for eligibility. Eligible participants will be presented with the Informed Consent and recruited (30 participants). Clinical, neuropsychological, neurophysiological and neuroimaging assessments will be performed before (T pre) and after (T post) the intervention

The DiSCIoser trial is designed as a randomized, controlled, assessors blinded single-centre trial with 2 parallel groups with 1:1 allocation ratio. The study flow is illustrated in Fig. 1. The participants recruitment, intervention delivery and data collection will take place at the Spinal Unit of FSL (Rome, Italy). Upon admission, patients with subacute cervical SCI will be screened for eligibility criteria by the project’s clinical staff (neurologists) according to the inclusion criteria of the study. Eligible participants will be introduced to the study protocol by authorized personnel, and they will be presented with the informed consent. Participants are randomized (within one week of first contact with the participants) to one of 2 intervention groups (as add-on): the BCI-MI training (experimental condition) or the Control-MI training (control condition).

The primary outcome is the Graded Redefined Assessment of Strength, Sensibility and Prehension (GRASSP) somatosensory sub-score [32]. Secondary outcomes are the GRASSP motor sub-score, the self-care section of the Spinal Cord Independence Measure (SCIM) [33] scale and a battery of clinical/functional scales (See Table 1 for details) to monitor upper limb muscle strength, tone, pain, and functional ability. Other pre-specified outcome measures are extracted from neuropsychological, neurophysiological and neuroimaging assessment before and after training. The overall outcome assessments are detailed in the Assessment section. The outcome assessors are blinded to the treatments.

**Table 1:**
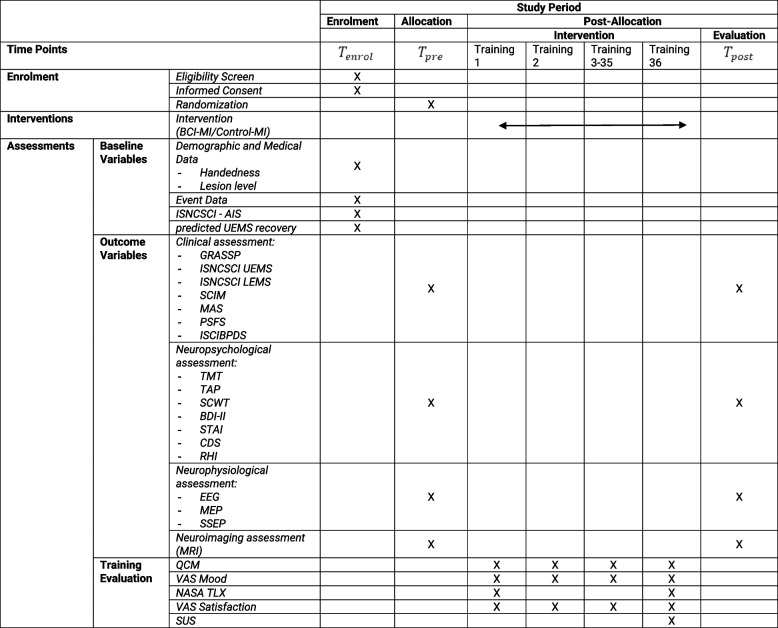
Standard protocol items as recommended for Interventional Trials (SPIRIT)

The study is presented according to the Standard Protocol Items: Recommendations for Interventional Trials (SPIRIT) [34]. Table 1 shows the SPIRIT schedule of enrolment, interventions, and assessments.

### Sample size

The sample size was calculated on the hypothesis that experimental intervention (BCI-MI training) is superior in improving the primary outcome measure. Based on preliminary findings in the Upper Extremity Motor Scores (UEMS) from the International Standards for Neurological Classification of SCI (ISNCSCI) ASIA Impairment Scale (AIS) [35] in experimental vs control condition, alpha level at 0.05, statistical power at 80%, and one-tailed t-test, 15 participants per group (Control-MI and BCI-MI) are needed. The analysis was performed using the statistical software STATISTICA 8.0 (Stat Soft. Inc. 1984–2007, USA) according to the algorithm implemented in the tool Power Analysis/Sample Size Calculation.

### Inclusion/exclusion criteria

The specific Inclusion and Exclusion criteria are listed below:

Inclusion criteria:

- age between 18 and 70 years,
- current hospitalization at FSL site for rehabilitation care,
- subacute traumatic cervical SCI (30–90 days after the event),
- classification according to ISNCSCI ASIA Impairment Scale A-D,
- lesion level between C1 and T1,
- predicted average UEMS recovery of less than 40/50 [36].

Exclusion criteria:

- other conditions (present or previous) potentially affecting sensorimotor function of the upper extremities,
- significant traumatic brain injury,
- history of epilepsy or previous neurological deficits,
- psychiatric or cognitive comorbidities involving motor planning problems or impulsivity (unable to follow commands),
- inability to give informed consent and understand training requirements,
- concurrent enrolment in other clinical research trials focusing on specific unconventional treatments targeting upper limb function.

### Assessments

#### Outcome assessments

Outcome measures refer to the assessments that will be conducted both before and after intervention. The evaluation timepoints are:

${T}_{pre}$ at randomization, before intervention.

${T}_{post}$ at the end of the intervention.

(See the SPIRIT diagram in Table 1 for details).

Trained clinical/research staff neurologists, neuropsychologists, neurophysiologists, therapists, and radiologists will perform the assessments and will be blind to the participant intervention allocation except for recruiting neurologists who are not blinded and do not participate to the assessment. Data will be recorded in an ad hoc Case Report Form (CRF; details in Data collection and management section).

#### Primary

Changes from pre- $({T}_{pre})$ to post- $({T}_{post})$ treatment in GRASSP somatosensory scores of bilateral arms (12 is the maximum score for each side, equal to physiological condition) [32] are the primary outcome measure of the trial. Those changes will be computed assessing the difference between ${T}_{post}$ and ${T}_{pre}$ scores, or by computing the effectiveness, defined as the ratio between ${T}_{post}$-${T}_{pre}$ difference and the maximum possible improvement for that specific scale, expressed in percentage.

#### Secondary

The secondary outcome measures are the changes from pre- $({T}_{pre})$ to post- $({T}_{post})$ of the following fields and assessment scales:

- muscle strength: the GRASSP motor score of bilateral arms that ranges from 0 (maximum impairment) to 50 (normal) for each side [32]; the UEMS and the Lower Limb Motor Score (LEMS) from the ISNCSCI AIS evaluation for the residual strength assessment in upper and lower limb segments, it ranges from 0 to 25 for each arm and leg [35]; the arm will be considered as dominant and non-dominant according to the results of the Edinburgh Handedness Inventory [37];
- muscle tone: the Modified Ashworth Scale (MAS) for the muscle tone assessment of upper and lower limbs [38] ranging from 0 (absence of augmented muscle tone) to 4 (non-reducible spasticity); the Penn Spasm Frequency Scale (PSFS) for the self-assessment of upper and lower limb spasms frequency (ranging from 0, absence of spasms, to 4, more than 10 spasm each hour) and severity (ranging from 1, mild, to 3, severe) [39];
- pain: the International SCI Pain Basic Dataset (ISCIBPDS) for pain assessment; a pain-intensity, rating from 0 to 10, quantifies pain impact on activities daily living, humour, and sleep functions [40];
- functional ability: the GRASSP prehension ability ranging from 0 to 12 for each side and prehension execution ranging from 0 to 20 for each side; the Spinal Cord Injury Independence Measure (SCIM) self-care section score ranges from 0 (maximum impairment) to 20 (independence) [33].

Other specified secondary outcome measures will be extracted from neurophysiological, neuroimaging, neuropsychological and user experience assessments (see below).

##### Neurophysiological assessment

This includes:

- the Motor Evoked Potentials (MEPs) to evaluate the changes in corticospinal tract integrity. MEPs will be recorded from the bilateral Abductor Digiti Minimi (ADM, for the upper limbs) and Abductor Hallucis (AH, for the lower limbs) muscles by following the methodology reported in [41]. In brief, transcranial magnetic stimulation will be delivered in single stimuli at maximum output stimulator (5 stimuli for each side) and electromyographical (EMG) activity will be recorded from ADM/AH muscle bilaterally, if allowed during voluntary contraction. EMG traces will be superimposed to determine MEP latency. Peripheral motor conduction time (PMCT) will be obtained by recording Compound Muscle Action Potentials (CMAP) and F-waves from ulnar and tibial nerves (upper and lower limbs respectively) bilaterally. PMCT will be calculated as (CMAP latency + F wave latency – 1)/2. Central motor conduction time will be calculated as MEP latency – PMCT.
- the Somatosensory Evoked Potentials (SSEPs) to evaluate the ascending sensory pathways. SSEPs will be recorded according to clinical standards, as briefly reported below. Electrical stimulation will be delivered to the median (upper limbs) and tibial (lower limbs) nerves (at motor threshold or 4 times sensory threshold, when applicable). Responses will be recorded at the Erb point (N9), cervical cord (N13) and scalp (N20) for the upper limbs and at lumbar cord (N22) and scalp (P40) for the lower limbs via bipolar derivations by means of surface electrodes, bandpass filtered between 30–3000 Hz and averaged (at least 2 averaged responses of minimum 100 stimuli each, according to patients’ collaboration and recording conditions – signal to noise ratio) [42].
- the high-density EEG recordings to evaluate the neurophysiological substrates of the experimental intervention efficacy at ${T}_{pre}$ and ${T}_{post}$ and to identify offline the BCI control features for each participant, see Intervention - *BCI-assisted motor imagery training (experimental intervention)* section. All participants will be comfortably seated in an armchair (or directly on their wheelchair) in a dimly lit room (laboratory) with their upper limbs resting on a pillow. EEG data will be collected from 61 positions, assembled on an active electrode cap (g.GAMMAcap equipped with g.SCARABEO active electrodes, g.tec medical engineering GmbH Austria) according to an extension of the 10–20 International System; ground: left mastoid, reference: auricular lobes. EMG data will be collected from the extensor digitorum and flexor digitorum superficialis muscles of both upper limbs and from the extensor indicis proprius of the dominant arm. Biosignals will be recorded and amplified by the g.HIamp amplifier (g.tec medical engineering GmbH Austria).
  Each recording session will consist of (i) four minutes of EEG recording at rest (closed and opened eyes), (ii) four runs during the MI tasks (MI session), (iii) four runs during the finger tapping task (FT session) whether the participant can perform the task.
  Each MI session will consist of 4 runs, 40 trials each. Each run will consist of 2 tasks: i) hand kinesthetic MI [43] task which will consist of either hand closing or opening, (identical to those performed during the intervention training) and it will involve simultaneously both hands and, ii) rest. Each run will include 20±1 MI trials and 20±1 rest trials in a randomized order. At the beginning of the session, participants will be allowed to attempt the execution of the motor tasks with both hands (whenever possible) for a limited number of trials to facilitate task comprehension and the rehearsal of sensorimotor sensations evoked by the actual execution during MI performance. Instructions for the participant will be “Imagine to close/open your hands in first person by trying to feel the sensations as if you were actually performing the movement”. Visual cues will be presented as visual representation of two hands on a black screen, dimly illuminated for 3s, then both hands will be lit-up and participants will be instructed to imagine the hands movement for 4s, then both hands will disappear from the screen when the trial ends. In rest trials, both dimly lit hands will be presented to the participants for the whole trial duration (7s) after which a black screen will appear. Each trial will be contextually defined by a written instruction on the screen (“rest” “image hands closing” “image hands opening”) and verbal cues from the researcher. During the intertrial interval, participants will be confronted with a black screen. The total trial duration will be 8.5s with an intertrial interval of 1.5s. EEG and EMG data will be recorded and sampled at 256 Hz.
  Changes of the motor relevant oscillatory activity will be measured as significant changes in the Power Spectral Density (PSD) maps (details in [30]). The PSD analysis will be performed offline on the high-density EEG data recorded at ${T}_{pre}$ and ${T}_{post}$ to describe the differences between the BCI-MI and Control-MI conditions. Changes of the brain networks between ${T}_{pre}$ and ${T}_{post}$ will be also measured as significant changes in the EEG-derived functional connectivity under rest (resting state) and task conditions (MI with both hands) [44–46].
  As for the FT, the session will consist of 4 runs, 30 trials each. In each trial the participant will attempt the execution of the index finger tapping movement with his dominant hand assessed as in [37] . Before the experiment, the participant will have a few minutes to familiarize themselves with the task. Instructions for the participant will be “Attempt to execute the index finger tapping as quickly and widely as you can”. Visual cues will be presented to the participant: a fixation cross lasting 2 s will forerun the beginning of each trial. Participants will be instructed to perform the finger tapping when a green circle will appear on the screen (stimulus duration of 0.8 s) and rest during the black screen (resting duration randomly set in the range from 3 s to 7 s). EEG and EMG data will be recorded and sampled at 1024 Hz. EEG data will be pre-processed and aligned according to the movement onset extracted by the EMG activity recorded over the extensor indicis proprius muscle and analysed to extract the movement related cortical potentials, well-known as descriptors of the impairment in SCI population [47].

##### Neuroimaging assessment

The neuroimaging protocol is derived from Cohen-Adad et al. [48]. For registration purposes, T_1_- and T_2_-weighted volumetric images will be acquired to assess lesions and to compute the spinal cord Cross-Sectional Area (CSA) with a sagittal orientation along the spinal cord. Protocol will be based respectively on MPRAGE, 1^3^ mm^3^ voxels, FOV 320 × 260 mm^2^, TE/TI/TR = 3.72/1000/2000 ms, and SPACE, 0.8^3^ mm^3^ voxels, FOV 256 × 256 mm^2^, TE/TR = 120/1500 ms. Magnetization Transfer (MT) imaging will be performed to compute MT parameters, with Gradient Echo scanning, pseudo-axial slices oriented transversally to the spinal cord, 0.9 × 0.9x5 mm^3^ voxels, FOV 230 × 230 mm^2^. Multi-Gradient-echo images will be collected pseudo-axially to compute grey matter and white matter CSA with 0.9 × 0.9x5 mm^3^ voxels, FOV 224 × 224 mm^2^, TE_min_/TR = 14/600 ms, 3 combined echoes. Diffusion-weighted images will be acquired pseudo-axially to compute diffusion metrics, exploiting an inner-excitation spin-echo EPI sequence, 0.9 × 0.9x5 mm^3^ voxels, FOV 86 × 32 mm^2^, TE/TR = 62/3600 ms, b-values 0 s/mm^2^ (6 scans) and 550, 1000 s/mm^2^ along 63 isotropic directions.

All scans will be performed at FSL Neuroimaging Unit on a high-performance 3 T scanner (Siemens Magnetom Prisma) equipped with a 64-channel head and neck coil and a 32-channel spine array, after 2^nd^ order shimming where appropriate.

Scans will be collected in both experimental and control groups at ${T}_{pre}$ and ${T}_{post}$ and will allow comprehensive characterization of lesion features and of tissue microstructural integrity.

##### Neuropsychological and user experience assessment

The Neuropsychological assessment at ${T}_{pre}$ and ${T}_{post}$ includes the Trail Making Test (TMT) [49], the Test for Attentional Performance (TAP) [50] considering alertness, Go/no Go and working memory subtests, the Stroop Color and Word Test (SCWT) [51] to assess executive function and cognitive flexibility, the Beck Depression Inventory II (BDI-II) [52] to investigate the presence and severity of depression symptoms and the State Trait Anxiety Inventory (STAI) [53] to assess state and trait anxiety. The assessment also includes the Rubber Hand Illusion task (RHI) [54], a paradigm to assess the illusory ownership of a fake hand as part the body following synchronous tactile stimulation over a visible rubber hand and the hidden real hand of the subject, and the Cambridge Depersonalization Scale (CDS) [55] which is a scale used to assess the frequency and duration of depersonalization symptoms.

Finally, the user experience assessment of the technology-based training [56] will include:

- at each single training session: before starting an adapted version of the Questionnaire for Current Motivation (QCM) [57] to evaluate the motivation and the Visual Analog Scale (VAS) [58] ranges from 0, “very bad mood”, to 10, “very good mood” to monitor the patient’s mood; after the session the VAS will be used for evaluating the satisfaction defined as “freedom from discomfort and positive attitudes toward the use of the product”: it ranges from 0 “not satisfied” 10 “absolutely satisfied;
- at the first and last training session: the National Aeronautics and Space Administration Task Load Index (NASA-TLX) [59] for the workload related to the training;
- at the end of last training session: the System Usability Scale (SUS) [60] for usability, acceptability and satisfaction of technology evaluation.

### Randomization procedure and methods

The random allocation sequence of participants to experimental (BCI-MI training) or control (Control-MI training) intervention groups will be generated by using Matlab (The MathWorks Inc, Natick, Massachusetts, USA, release 2019a).

The randomization sequence will be stratified by age (2 categories: ≤ 50 years old and > 50 years old) [61] and by GRASSP somatosensory scores (2 categories: ≤ 8 and > 8). A 1:1 allocation ratio will be used. For each stratum, the sequence will include 30 random allocations. The allocation sequence is securely stored at FSL. Randomization of participants will stop after randomization of the 30th participant in the study by summing participants in all strata.

### Intervention

#### Dosage of intervention training

Both BCI-MI and Control-MI training will be completed in 12 weeks with a weekly frequency of 3 sessions per week, lasting 45 min. Treatment adherence is set at 12 of the 36 intervention training sessions. We do not expect the BCI-MI and Control-MI interventions to cause adverse effects (non-invasive procedure; no drug-administration; no adverse events [30]) and both intervention deliveries will be under the care of SCI specialized personnel (physiotherapists, neurophysiology technician experts in patient EEG recordings). To monitor any possible deviation from the intervention training protocols (e.g., missing sessions for intervening illness), a report form for each participant will be compiled at each planned training session by the training personnel and it will be part of the CRF (CRF- Training section; details in Data collection and management section). The study interventions are conceived as add-on to the standard rehabilitation care (treatment as usual; TAU), and both groups will receive the same dosage of TAU and add-on training. To ensure comparability between and within group, TAU will be delivered according to an equal intensive regimen for both groups. The intensive regimen includes neuromotor physiotherapy sessions of 40 min each, twice a day, except on Saturdays, when it is once a day, for 6 days a week.

#### BCI-assisted motor imagery training (BCI-MI, experimental intervention)

**Fig. 2:**
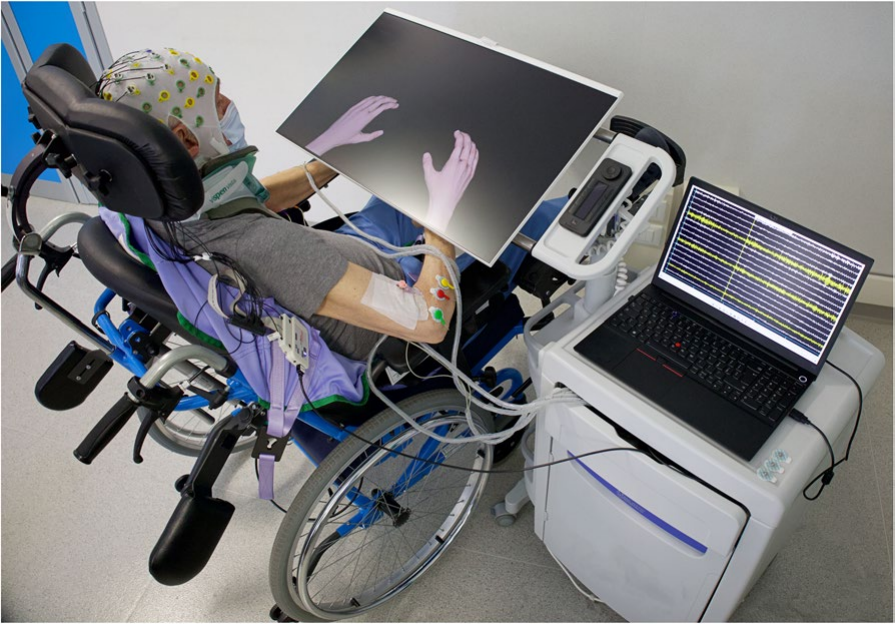
DiSCIoser BCI station. The Brain-Computer Interface training station developed for patients with Spinal Cord Injury to practice the kinaesthetic motor imagery of hand closing and opening in a close-loop condition. The system is equipped with a laptop, a commercial wireless EEG/EMG system and a screen for the ecological feedback to the participant. The ecological feedback is delivered by means of a custom software program that provides for (personalized) visual representation of the participant’s own hands. The screen for the feedback is adjustable in height and tilt to adapt to use in wheelchair or bed. The participant in the photo filled out the photo release consent form

An all-in-one BCI-supported MI training station (Fig. 2) developed considering the requirements and specific needs of patients with cervical SCI, will allow to practice the kinesthetic MI of hand closing and opening in a closed-loop condition. To further engage patients during the MI training, and to make it more appealing, the BCI station also includes the option of performing the MI of goal-oriented movements. For example, the hand opening or closing will allow the patient to burst soap bubbles, move billiard balls, and turn on/off light bulbs within a virtual environment. Therapists will choose on a session-by-session basis whether to use animation and also which type in order to introduce variation in the training. The type of animation used in each session will be recorded on the specific training CRF.

The feedback that participants receive, i.e., their own hand opening or closing in the virtual environment, will be controlled by the BCI control features. They are those relevant, significant frequency/spatial changes of the EEG signals related to the MI task. The control features will be extracted through offline analysis of the MI-related EEG data collected at ${T}_{pre}$ (see Neurophysiological Assessment).

A semiautomatic, physiologically driven EEG feature selection method, GUIDER algorithm [62], will be used to process EEG data. Briefly, the GUIDER algorithm permits the user to spatially filter EEG data by means of the common average reference filter, divide EEG data into 1 s-length epochs and extract all spectral features (i.e., spectral amplitude at each frequency bin for each channel) in a reasonable range (0-36 Hz) for each epoch using maximum entropy method (16th order model, 2 Hz resolution, no overlap). A priori criteria for the selection of the relevant control features are then applied: i) features are derived from the fronto-central and centroparietal electrodes that are distributed over both hemispheres; ii) they show desynchronization patterns i.e., a decrease in spectral power at EEG frequencies that are typical for the modulation of sensorimotor rhythms induced by the MI tasks. Thus, through BCI training, we aim to reinforce the individual EEG patterns of reactivity that most resembles the physiological activation that is relevant to the movement imagination of both hands. Qualified neurophysiologists will be instructed to use the GUIDER tool and will flag for each single participant the EEG channels over the scalp sensorimotor areas to be included in the analysis. GUIDER will then extract (regression modeling) the optimal subset of predictor variables that best discriminate between the dependent variables (e.g., task *vs* rest) and it will return an external parameter file ready to be loaded on BCI2000 software [63] for the training sessions.

#### Motor imagery training without BCI support (Control-MI, control intervention)

The Control-MI training will be implemented under the same conditions as the experimental intervention (BCI-MI training). Specifically, the station (Fig. 2) will be used to provide participants only with a visual cue of the MI trial duration that is, the visually represented hand stands still with no BCI control. Participants will perform the same MI tasks (opening/closing hand trial, in random order) as in the experimental intervention training.

#### Training sessions

During training sessions, all participants will be seated on a comfortable chair (or on their wheelchair) in a dimly lit room with their hands and forearms resting under the BCI station screen (Fig. 2). Alternatively, if necessary, it will be possible for the patients to perform the session in bed in a semi-sitting position with hands and forearms resting under the BCI station screen.

Participants will be instructed by the therapists to perform the kinesthetic MI of both hands movements either closing or opening in separate runs randomly ordered. Each training session will consist of 4 runs (20 trials each). In the case of BCI-MI training, each trial will consist of constant baseline period of 4 s and MI task period of maximally 10 s in case of unsuccessful trial, whereas for the Control-MI training the trial length is fixed and randomly choose in the range 4-6 s. In both intervention trainings, EEG data will be collected from 16 EEG electrodes, assembled on an electrode cap according to an extension of the 10–20 International System (ground: left mastoid). Electrode positions will cover the bilateral sensorimotor area (FC3, FC1, FCz, FC2, FC4, C3, C1, Cz, C2, C4, CP3, CP1, CPz, CP2, CP4, Pz). For each participant in BCI-MI group the individual significant EEG features (extracted as described in *BCI-assisted motor imagery training)* section will serve as BCI control features. EMG activity will be continuously recorded through surface electrodes placed over the extensor and flexor digitorum muscles of both hands to ensure that no actual movement is performed during training. EEG and EMG data will be digitalized at 250 Hz (LiveAmp, Brain Products GmbH, Germany).

### Statistical analysis

#### Primary analyses

Baseline characteristics will be described by summary statistics for each group (experimental and control intervention). Differences between groups at baseline ${T}_{pre}$ will be tested to check that the groups do not differ statistically at baseline.

The primary analysis will be performed in per protocol (PP) population on GRASSP somatosensory score changes between ${T}_{pre}$ and ${T}_{post}$. The PP will include all randomized participants who will perform (minimum) 12 training sessions. T-test or Wilcoxon Mann–Whitney rank test for independent groups will be used to compare continuous and discrete outcome measures respectively. Statistical significance will be assessed by two-tailed test.

#### Secondary analyses

All secondary outcomes will be compared between groups. T-test for independent samples and non-parametric tests will be used for continuous and categorical data, respectively. Further explorative analyses will be carried out on primary and secondary outcomes in subgroups of participants identified by strata used in randomization procedure. The statistical methods adopted for primary and secondary analyses will be used. All analyses will be under the expert supervision of the partner SAP.

### Data collection and management

An ad hoc CRF is implemented for all type and timing assessments.

Specifically, the CRF will consist of two sections:

i) the “Baseline and Randomization Section” which will contain each participant demographic, neurological and clinical data and all data collected for the screening section including the Informed Consent, the assigned randomized treatment, and the assessment before intervention (${T}_{pre}$). This part will be filled in by unblinded personnel.

ii) the “Outcome and Training Section” which will not include data on assigned experimental treatment but will contain all the outcome collected in each training session and during the assessment after intervention (${T}_{post}$). It will be filled by blinded personnel, namely the outcome assessors.

A specific standard operating procedure including time schedule, and instruction for management and compilation of the CRF will be used. All study staff responsible for outcome assessment (neurologists, neuropsychologists, neurophysiologists, therapists, neuroimaging researchers and other researchers involved) and training (therapists and EEG technicians) will be trained on procedures to ensure validity and reliability of trial data collection.

### Confidentiality

Confidentiality and Privacy will be managed according to Italian National Law.

Personal data are regarded as strictly confidential. Original paper CRFs containing study data are stored at FSL and subjected to all GDPR (UE 2016/679) security regulation and backup as clinical/medical records. Data are entered using participant unique study codes (pseudo-anonymization). The access to all study files is restricted to FSL staff involved in the study. The BCI station system records EEG/EMG data from each participant for each training session; data will be stored by unique study code only.

### Trial monitoring

Database collection within and between Experimental and Control intervention group will be monitored and the trial responsible will be alerted if any deviation occurs. Any modifications to the protocol which may impact on the conduct of the study, including changes of study objectives, study design, participant population, sample sizes, study procedures, or significant administrative aspects will require a formal amendment to the protocol. Such amendment will be agreed upon by the principal investigator and approved by the Ethics Committee prior to implementation and notified to the National Ministry of Health (sponsor).

### Dissemination of results

Main results will be subjected to publications in scientific peer-reviewed journals; results will be also presented at clinical neuroscience and/or Neuroengineering (Society for Neuroscience conference; BCI international conference; IEEE; National Society for Neurorehabilitation) conferences. Media and public outreach are planned.

## Discussion

Traumatic cervical SCI may lead to long-term disability for which cost-effective rehabilitation options are critically needed. The DiSCIoser study aims at providing evidence for the clinical/neurophysiological efficacy of BCI-based intervention to promote meaningful cortical sensorimotor plasticity and eventually maximize recovery of arm functions in subacute cervical SCI. This study will generate a body of knowledge that is fundamental to drive optimization of BCI application in SCI as a top-down therapeutic intervention (taking advantage of brain plasticity), thus beyond the canonical BCI use as assistive tool (i.e., neuroprosthetic controller).

## Data Availability

Not applicable.

## Abbreviations

ADM: Abductor Digiti Minimi
AH: Abductor Hallucis
AIS: ASIA Impairment Scale
BCI: Brain-Computer Interface
BDI-II: Beck Depression Inventory II
CDS: Cambridge Depersonalization Scale
CMAP: Compound Muscle Action Potentials
CRF: Case Report Form
CSA: Cross-Sectional Area
EEG: Electroencephalography
EMG: Electromyography
FSL: Fondazione Santa Lucia
FT: Finger Tapping
GRASSP: Graded Redefined Assessment of Strength, Sensibility and Prehension
ISNCSCI: International Standards for Neurological Classification of SCI
ISCIBPDS: International SCI Pain Basic Dataset
LEMS: Lower Limb Motor Score
MAS: Modified Ashworth Scale
MEP: Motor Evoked Potentials
MI: Motor Imagery
MRI: Magnetic Resonance Imaging
MT: Magnetization Transfer
NASA- TLX: National Aeronautics and Space Administration Task Load Index
PDC: Partial Directed Coherence
PMCT: Peripheral motor conduction time
PP: Per Protocol
PSD: Power Spectral Density
PSFS: Penn Spasm Frequency Scale
QCM: Questionnaire for Current Motivation
RCT: Randomized Controlled Trial
RHI: Rubber Hand Illusion task
SAP: Sapienza University of Rome
SCI: Spinal Cord Injury
SCIM: Spinal Cord Independence Measure
SCWT: Stroop Color and Word Test
SPIRIT: Standard Protocol Items: Recommendations for Interventional Trials
SSEP: Somatosensory Evoked Potentials
STAI: State-Trait Anxiety Inventory
SUS: System Usability Scale
TAP: Test for Attentional Performance
TAU: Treatment as usual
TMT: Trail Making Test
UEMS: Upper Extremity Motor Score
VAS: Visual Analog Scale
